# Evolutionary Biology at Belyaev Conference – 2017

**DOI:** 10.1186/s12862-017-1102-0

**Published:** 2017-12-28

**Authors:** Yuriy L. Orlov, Ancha V. Baranova, Yuriy E. Herbeck

**Affiliations:** 1 Institute of Cytology and Genetics SB RAS, Novosibirsk, Russia; 2 Novosibirsk State University, Novosibirsk, Russia; 3 George Mason University, Fairfax, USA; 4 Research Centre of Medical Genetics, Moscow, Russia

Current collection continues the series of BioMed Central special post-conference issues presenting the highlights from the set of conferences on bioinformatics and systems biology held in Novosibirsk and Moscow (Russia) in 2017. This thematic issue of BMC Evolutionary Biology issue cover the papers presented at Young Scientists School “Systems Biology and Bioinformatics - 2017” (SBB-2017) and Belyaev memorial conference “Belyaev Readings - 2017” (BR-2017). Previously published special issues of *BMC Evolutionary Biology* and *BMC Genomics* covered the proceeding of BGRS\SB-2016 conference and SBB-2015 School in Novosibirsk [1–4] as well as BGRS\SB-2014 event (https://bmcgenomics.biomedcentral.com/articles/supplements/volume-15-supplement-12).

In it important to note that Year 2017 marks the 100th anniversary since birth of Full Member of the USSR Academy of Sciences, Professor Dmitry K. Belyaev (1917–1985), an outstanding scientist, evolutionist and geneticist. In view of this memorable date, the Institute of Cytology and Genetics of the Siberian Branch of the Russian Academy of Sciences (ICG SB RAS) held international Belyaev Conference on Genetics and Evolution (Novosibirsk, August 7–10, 2017 - http://conf.bionet.nsc.ru/belyaev100/en).

Back in 1950s, Russian (Soviet) geneticist Dmitry Konstantinovich Belyayev embarked on a journey to turn aggressive, wily silver fox into a domestic animal as friendly and tail-wagging as a dog. Belyaev was inspired by Darwin’s early observations on similarity of morphologic and physiologic changes observed in various domestic animals. Hence, he hypothesized that domestication is, in fact, a selection for disposition toward humans and, therefore, it should involve the changes in regulatory genes governing the behavior, the stress and the metabolism of sex hormones. As a result, a unique, world-famous tame fox population was generated. Belyaev demonstrated that the selection for human-friendly demeanor disrupts physiological and morphological systems of silver fox, thus, shifting the phenotype to that similar to the dogs and other now-tamed animals. With that, Belyaev opened up a totally new chapter in the theory of morphogenetic processes that explores interactions between two main factors in evolution: variability and selection. The results of a breathtaking experiment with thousands of animals led Belyaev to postulate a very special form of directional, destabilizing selection taking place in domestication. It destabilizes regulatory systems underlying ontogenesis and, as a consequence, sharply increases the rates at which new phenotypic forms emerge.

**Fig. 1:**
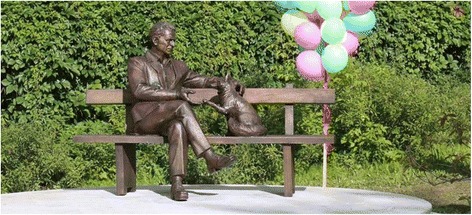
Photo: The opening of the monument to D.K. Belyaev and tame fox. August 2017, Novosibirsk

To highlight the significance of fox domestication experiments performed at the Institute of Cytology and Genetics SB RAS in Novosibirsk, the unique bronze monument to Academician D. K. Belyaev was built by collective efforts near the Institute, where he served as a Founding Director (http://icg.nsc.ru/belyaev100/en/monument/). The tamed fox gives the Professor a paw and wags the tail (Fig. 1).

In 2017, “Vavilov Journal of Selection and Breeding” published a series of memoirs publications about Prof. Belyaev (http://vavilov.elpub.ru/jour/issue/view/32/showToc). The article by Prof. V. K. Shumny [5] tells the history of Belyaev’s life, while other publications discuss importance of Belyaev’s work on the theory of evolution and domestication [6, 7].

Current issue of *BMC Evolutionary Biology* presents works describing evolutionary insights derived from observations and experiments with a range of lifeforms as large as the whales and as small as the microbes, uncovering novel adaptations.

Alexey A. Moskalev and co-authors [8] consider the problems of aging and longevity through the prism of marine biology. In their study, they assembled genome and transcriptome of a grey whale, which is often described as a “living fossil”, adapted to extreme marine conditions. This work puts a cornerstone for further studies of whale evolution and longevity.

The work of Nataly E. Gruntenko and colleagues [9] consider the response to parasite-induced stress in a classical model of fruit fly. This team showed that the strains of Wolbachia differ in their respective influence towards dopamine metabolism of Drosophila and their survival under heat stress conditions.

Nadezhda L. Bolsheva and co-authors [10] presented their work in the area of plant evolution. After analysis of ribosomal RNA genes in various species of the blue-flowered flaxes (genus *Linum*), this research team made several adjustments to the phylogeny of these plant species, while also showing both intra- and interspecific divergence of their rRNA-encoding genes.

Alexey S. Rozanov et al. [11] describe biodiversity of microbial organisms living in extreme natural conditions within the microbial mats of alkaline hot spring Garga within the river Barguzin of the Baikal rift zone. They showed a high abundance of novel species of Archaea and the domains of heterotrophic organisms. Studied microbial mats had evolved in early stages of biosphere formation.

Elena V. Ignatieva [12] and colleagues discuss problems of human response to tick-borne encephalitis virus infection and reconstruct the networks of human genes involved in such response. In their article evolutionary problems of human adaptation to parasites and infections are discussed.

Follow-on series of related works in the areas of classical and medical genomics, genetics, and plant biology discussed at “Belyaev conference – 2017” and other related meetings in Novosibirsk and Moscow will be published in Special Issues of *BMC Genetics, BMC Genomics, BMC Medical Genomics, BMC Plant Biol* and *BMC Neuroscience*.
